# Relationship of anti-SARS-CoV-2 IgG antibodies with Vitamin D and inflammatory markers in COVID-19 patients

**DOI:** 10.1038/s41598-022-09785-7

**Published:** 2022-04-05

**Authors:** Hatixhe Latifi-Pupovci, Sadie Namani, Artina Pajaziti, Blerina Ahmetaj-Shala, Lindita Ajazaj, Afrim Kotori, Valdete Haxhibeqiri, Valentin Gegaj, Gramoz Bunjaku

**Affiliations:** 1 University of Pristina, Georg Bush, No.31, 10000 Prishtina, Kosovo; 2 University Clinical Center of Kosovo, Pristina, Kosovo; 3 National Heart and Lung Institute, Imperial College London, London, UK; 4 University for Business and Technology, Pristina, Kosovo

**Keywords:** Biomarkers, Medical research

## Abstract

Several studies have found an association of COVID-19 disease severity with Vitamin D deficiency and higher levels of anti-SARS-CoV-2 IgGs. The aim of this study was to determine whether levels of Vitamin D and “inflammatory state” influence the magnitude of anti-SARS-CoV-2 IgGs levels in COVID-19 patients. For this purpose, in 67 patients levels of anti-SARS-CoV-2 IgG were measured in week 4 whereas in 52 patients levels of Vitamin D were measured in week 1 after symptom onset. We found that low Vitamin D levels were significantly associated with age and disease severity whereas there was a trend without significance, towards negative correlation of Vitamin D with anti-SARS-CoV-2 IgG. Anti-SARS-CoV-2 IgG were significantly higher in older ages, patients with severe disease, diabetes and those who received corticosteroid and antibiotic therapy. There was a positive correlation of anti-SARS-CoV-2 IgG with IL-6, CRP, LDH, ESR and with percentages of granulocytes. In conclusion, Vitamin D and anti-SARS-CoV-2 IgG share common parameters associated with inflammatory state. However, even though Vitamin D protects against severe forms of COVID-19 it could not directly affect anti-SARS-CoV-2 IgG production.

## Introduction

The coronavirus disease 2019 (COVID‑19) is caused by a new coronavirus which in addition to acute respiratory failure is associated with systemic disorders such as hyperinflammation, hypercoagulation and vasculitis^1^. Although many people exhibit mild ‘flu-like’ symptoms, in severe responses systemic changes have been attributed to the cytokine storm accompanying severe inflammatory syndrome^2–4^. Severe forms of COVID-19 have been linked with low levels of circulating 25-hydroxy Vitamin D (25[OH]D) as an expression of Vitamin D (Vit. D)^5–10^. Vit. D has immunomodulatory activity in response to invasion of bacterial and viral pathogens^11,12^ interacting with its receptor (VDR) in immune cells^13–15^. In several studies it was shown that severe inflammatory syndrome was accompanied with changes in hematological markers and increased several inflammatory markers such as CRP, LDH, ESR, ferritin etc.^2,16–18^. A recent study has shown that in the presence of Vit. D, IL-6 induces higher production of IL-10, a known anti-inflammatory cytokine which is expected to lead to the reduction of inflammatory markers such as CRP^19^. Several studies have shown inverse association between Vit. D and CRP levels^20–22^. Additionally, high levels of CRP were associated with lowering levels of Vit. D^23^ indicating that Vit. D is a negative acute phase reactant. Thus, Vit. D insufficiency could be the cause and effect of high CRP levels in COVID-19 patients.

Humoral and cellular immune responses, two wings of adaptive immunity, are crucial in clearing a variety of viral infections^24^, and have been implicated in recovered COVID-19 patients^25,26^. In several studies disease severity of COVID-19 was associated with higher levels of antibodies^27–29^ whereas asymptomatic patients minimally produced anti-SARS-CoV-2 IgGs which were poorly maintained^28,30^. Taking into account that Vit. D affects adaptive immune response, it may have an impact on serological response against SARS-CoV-2. The pathways on this impact could be through different molecules and cells^31–33^. Previous published data has demonstrated that Vit. D supplementation can boost antibody production after vaccination with Influenza virus^14^. Faniyi et al. showed that Vit. D deficiency (VDD) was an independent risk factor for COVID-19 seroconversion in healthcare workers^34^. Also, Kaufman et al. reported that COVID-19 positivity was inversely related to the patient’s Vit. D levels in the preceding 12 months^35^.

Knowing that till now there are a small number of reports on Vit. D effect in serological response in COVID-19 patients, the first aim of this study was to determine whether Vit. D and “inflammatory state” influence the magnitude of anti-SARS-CoV-2 IgG levels in COVID-19 patients. The second aim of the study was to analyze the change in Vit. D levels during the illness and the change in anti-SARS-CoV-2 IgG antibody levels after 3 months of disease onset.

## Results

A total of 69 patients diagnosed with COVID-19 were enrolled in this study. The median age of patients was 59 years (44.5–67.0 years) of whom 53.6% were females and 46.4% males. Patient characteristics are presented in Table 1.

**Table 1:** Patients characteristics.

		Patients (N = 69)	Patients with Vit. D-w1 (N = 52)	Patients with IgG-w4 (N = 67)
Sex N (%)	Female	37 (53.6)	26 (50.0)	37 (55.2)
	Male	32 (46.4)	26 (50.0)	30 (44.8)
Age	Median (IQR)	59.00 (44.50–67.00)	62.00 (51.50–70.00)	59.00 (44.00–67.00)
Hospitalisation N (%)	Yes	42 (60.9)	39 (75.0)	40 (59.7)
Disease severity N (%)	Mild	16 (23.2)	8 (15.4)	16 (23.9)
	Moderate	24 (34.8)	18 (34.6)	24 (35.8)
	Severe	25 (36.2)	22 (42.3)	25 (37.3)
	Critical	4 (5.8)	4 (7.7)	2 (3.0)
Comorbidities N (%)	Hypertension	27 (39.1)	22 (42.3)	27 (40.3)
	Diabetes Mellitus	10 (14.5)	9 (17.3)	10 (14.9)
	Cancer	5 (7.2)	5 (9.6)	4 (6.0)
	Hypothyreosis	4 (5.8)	2 (3.9)	4 (6.0)
Outcome N (%)	Recovery	65 (94.2)	48 (92.3)	65 (97.0)
	Death	4 (5.8)	4 (7.7)	2 (3.0)
Medications used during illness N (%)	Corticosteroids	49 (71.0)	43 (82.7)	47 (70.1)
	Antivirals	29 (42.0)	25 (48.1)	28 (41.8)
	Antibiotics	65 (94.2)	51 (98.1)	64 (95.5)
Vit. D Groups N (%)	Deficient (≤ 20)		17 (32.7)	15 (22.4)
	Insufficent (21–29)		17 (32.7)	17 (25.4)
	Sufficient (≥ 30)		18 (34.6)	18 (26.9)

### Age, disease severity and diabetes influence anti-SARS-CoV-2 IgG antibody levels

**Figure 1:**
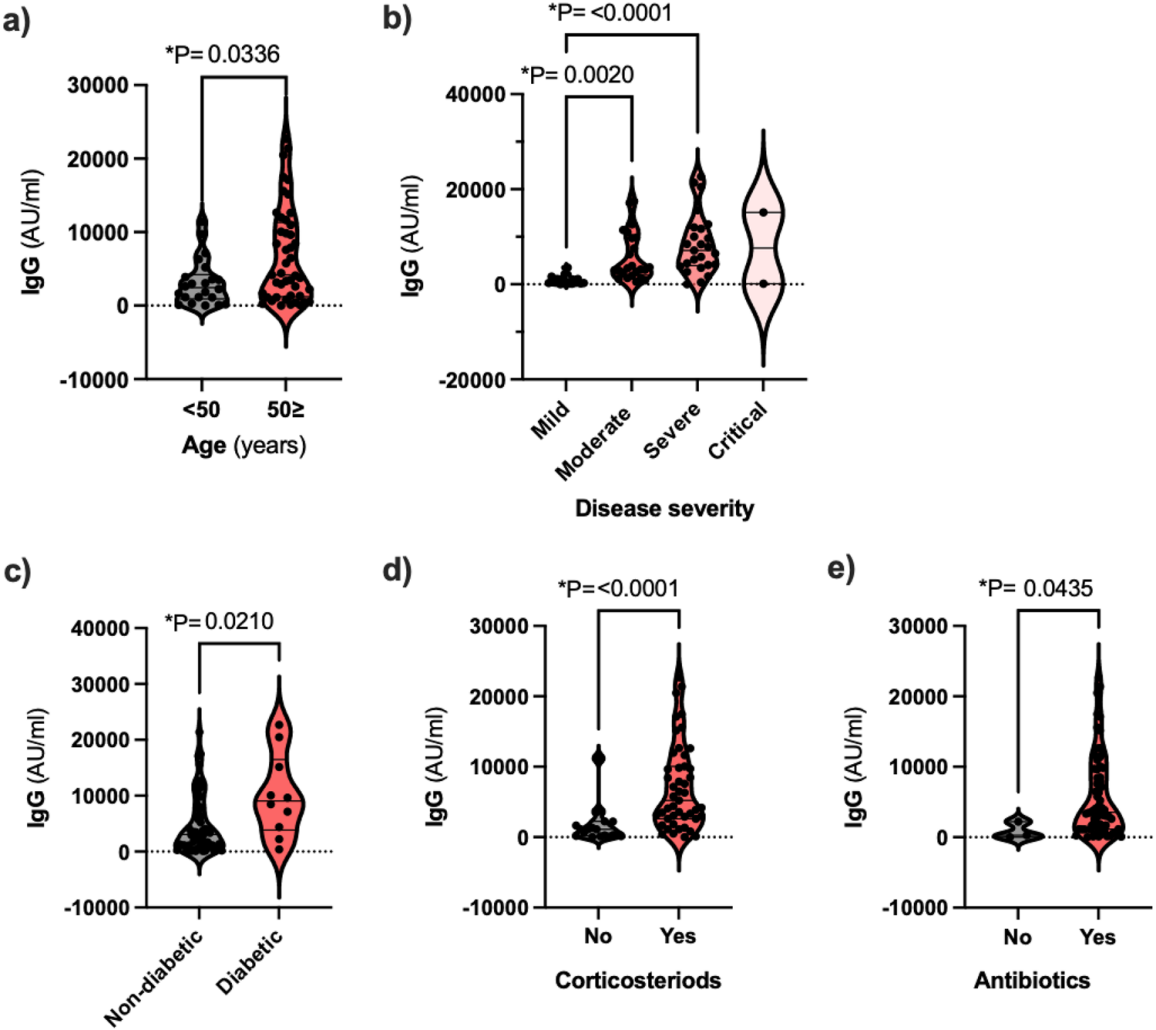
Distribution of anti-SARS-CoV-2 IgG across age groups, disease severity groups, patients with and without diabetes, corticosteroid and antibiotic administration. Levels of IgG were significantly higher in age group > 50, severe and critical patients, in diabetics and patients treated with corticosteroids and antibiotics.

We investigated the factors that are likely to influence anti-SARS-CoV-2 IgG levels in COVID-19 patients. We found that age, but not gender, significantly influenced IgG levels in this group of patients. Specifically, patients aged > 50 were found to have significantly higher IgG levels than patients < 50 (Fig. 1). When grouping according to disease severity the distribution of IgG-w4 was different across categories of disease severity. Mean ranks of anti-SARS-CoV-2 IgG were significantly higher in patients with diabetes and those who received corticosteroid and antibiotic therapy (Fig. 1). IgG levels were also higher in patients with hypertension but this did not reach significance (p = 0.054).

### Vit. D is associated with age and disease severity

**Figure 2:**
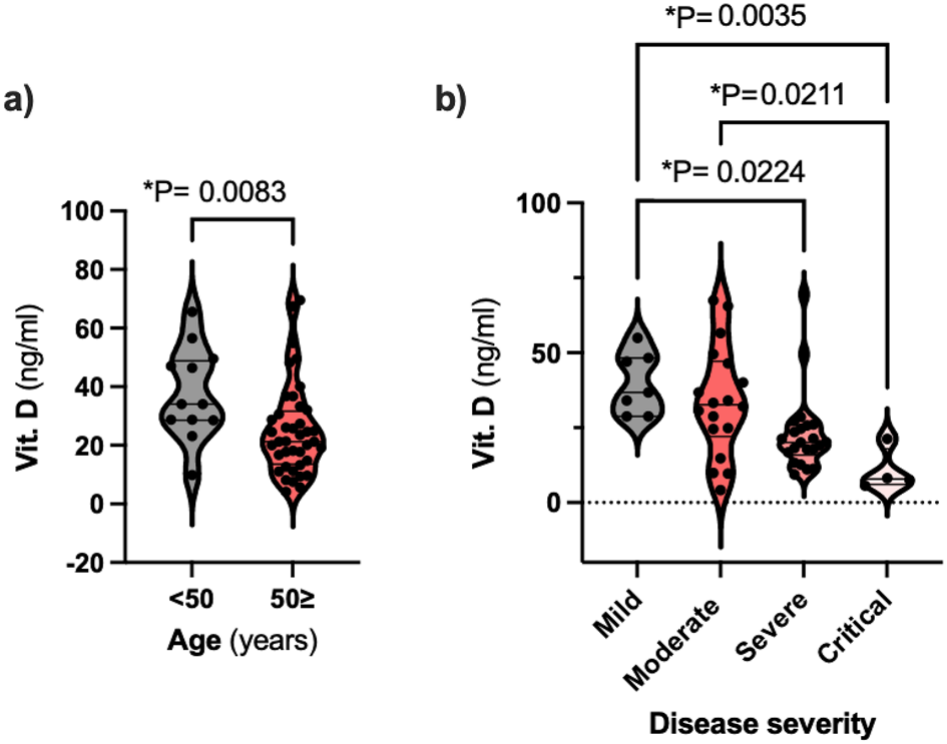
Distribution of Vit. D-w1 across age groups and disease severity groups. Levels of Vit. D were significantly higher in the age group < 50 and in mild/moderate patients.

Next, we sought to determine whether the magnitude of anti-SARS-CoV-2 IgG antibody level was related to Vit. D levels. In 65.4% of 52 patients, levels of Vit. D were below normal values. Gender did not influence Vit. D, however once again, age was significantly correlated with Vit. D. A significant difference in distribution of Vit. D was also found in different groups of patients according to disease severity and outcome (Fig. 2); all deceased patients were insufficient, 75% of them being VDD.

### Correlations between anti-SARS-CoV-2 IgG and Vit. D with other inflammatory and hematological parameters

To determine other factors that are likely to influence anti-SARS-CoV-2 IgG production and the relationship of Vit. D with these factors, we did a correlation analysis of anti-SARS-CoV-2 IgG and Vit. D with inflammatory and hematological markers in the first week post-symptom onset (PSO) and with the most changed values (maximum values of CRP, D-Dimer, LDH, ESR, percentages of granulocytes and minimum values of WBC count, platelet count, percentages of monocyte and percentages of lymphocytes) during the course of disease. Anti-SARS-CoV-2 IgG were positively correlated with IL-6, CRP, LDH, ESR and percentages of granulocytes determined in the first week PSO (Table 2). On the other hand, levels of IgG were weakly correlated with percentages of lymphocytes, but without significance. When analysed with the most changed values during the course of disease, we found stronger negative correlation between anti-SARS-CoV-2 with maximum values of CRP, LDH, ESR and percentages of granulocyte than with parameters found in the first week PSO. There are no maximum values of IL-6 for the fact that this parameter was determined only once, in the first week PSO. On the other hand, anti-SARS-CoV-2 resulted to be positively correlated with minimum values of WBC and negatively correlated with minimum values of percentages of lymphocytes and monocytes.

**Table 2:** Mutual correlation between IgG-w4 and Vit. D-w1 and correlation between these parameters and inflammatory and hematological markers.

	IgG-w4 (N = 67)			Vit. D-w1 (N = 52)		
	N	Spearman Rho	p value	N	Spearman Rho	p value
Vit. D-w1 (ng/ml)	50	− 0.151	0.296			
IL6 (pg/ml)	45	0.310	0.038	43	− 0.361	0.017
CRP-w1 (mg/l)	52	0.515	0.000	51	− 0.400	0.004
Max. CRP (mg/l)	63	0.539	0.000	51	− 0.486	0.000
D-Dimer-w1 (ng/ml)	50	− 0.018	0.903	49	− 0.290	0.043
Max. D-Dimer (ng/ml)	59	0.224	0.089	49	− 0.391	0.005
LDH-w1 (U/l)	25	0.515	0.008	25	− 0.295	0.152
Max. LDH (U/l)	28	0.560	0.002	25	− 0.097	0.645
CK-w1 (U/l)	20	0.242	0.304	21	− 0.124	0.591
Max. CK (U/l)	20	0.232	0.326	21	0.160	0.487
ESR-w1 (mm/h)	42	0.436	0.004	40	− 0.372	0.018
Max. ESR (mm/h)	47	0.552	0.000	40	− 0.385	0.014
WBC-w1 (× 10^9^/l)	57	0.241	0.071	52	− 0.131	0.354
Min. WBC (× 10^9^/l)	63	0.362	0.004	52	− 0.322	0.020
Granulocytes-w1 (%)	54	0.310	0.023	49	− 0.214	0.140
Max. granulocytes (%)	60	0.303	0.019	49	− 0.214	0.140
Lymphocytes-w1 (%)	55	− 0.236	0.083	50	0.159	0.271
Min. lymphocytes (%)	61	− 0.266	0.038	50	0.169	0.240
Monocytes-w1 (%)	44	− 0.250	0.101	39	0.120	0.466
Min. monocytes (%)	49	− 0.284	0.048	39	0.122	0.461
Platelet count-w1	56	− 0.132	0.332	51	0.372	0.007
Min. platelet count	61	− 0.123	0.346	51	0.415	0.002

Spearman correlation and significance.

When we analysed correlations between Vit. D and various inflammatory and hematological parameters, in the first week PSO we found a significant negative correlation between the Vit. D and IL-6, CRP, D-dimer, ESR and a significant positive correlation with platelet count. When we analysed correlation of Vit.D with the most changed laboratory parameters during the course of disease, stronger negative correlation was found between Vit. D and CRP, D-Dimer, ESR and platelet count than with these parameters in the first week PSO whereas a negative correlation was found also with minimum values of WBC count. There was a negative correlation of IgG-w4 with Vit. D-w1, however this did not reach statistical significance (p = 0.296) (Table 2).

### Changes in Vit. D and anti-SARS-CoV-2 IgG levels over time

In order to estimate the change of Vit. D during illness and the change of anti-SARS-CoV-2 IgG after 3 months, we compared median differences in paired samples (Vit. D-w1 and Vit. D-w4; IgG-w4 and IgG-m4). In 66% of patients (N = 50) levels of Vit. D decreased during the illness although all patients were supplemented with Vit. D; hospitalised patients were supplemented with 4000 UI/ml, whereas ambulatory patients took no more than 1000 UI/ml. There were no significant differences in Vit. D decrease during illness between groups of patients by sex, age, disease severity and comorbidity (Table 3).

**Table 3:** Vit. D level change (difference Vit. D-w1–Vit. D-w4) during illness and IgG level change 3 months PSO (difference IgG-w4–IgG-m4).

		Vitamin D change		IgG change	
		Difference Vit. D-w1–Vit. D-w4	Statistics	Difference IgG-w4–IgG-m4	Statistics
Frequency N (%)	Increase	17 (34.0)		11 (64.7)	
	Decrease	33 (66.0)		7 (35.3)	
Sex N (mean rank)			U = 266, p = 0.372		U = 30, p = 0.763
	F	26 (23.73)		11 (9.27)	
	M	24 (27.42)		6 (8.50)	
**Age N (mean rank)**			H(5) = 6.027, p = 0.304		H(4) = 4.036, p = 0.401
0–29		5 (25.9)		4 (8.50)	
30–39		3 (20.67)		3 (10.00)	
40–49		4 (41.88)		3 (8.67)	
50–59		10 (21.95)		3 (4.67)	
60–69		14 (24.89)		4 (12.25)	
70+		14 (24.86)			
**Disease severity N (mean rank)**			H(3) = 0.542, p = 0.910		H(2) = 6.489, p = 0.039
Mild		8 (25.31)		8 (6.63)	
Moderate		18 (24.00)		6 (9.00)	
Severe		22 (26.30)		3 (15.33)	
Critical		2 (31.00)			
Hospitalisation N (mean rank)			U = 194.5, p = 0.309		U = 2, p = 0.017
	No	13 (21.96)		14 (7.64)	
	Yes	37 (26.74)		3 (15.33)	
Difference between medians N (mean rank)			Z = − 2.129, p = 0.033		Z = − 2.107, p = 0.035
	Negative Ranks	33 (26.00)		11 (11.0)	
	Positive Ranks	17 (24.53)		6 (5.33)	

*U* Mann–Whitney U test, *H* Kruskal–Wallis test, *Z* Wilcoxon Signed Ranks test.

The changes of IgG levels between two measurements were analysed in 17 patients. In 6 patients we found an increase of IgG levels, whereas in 11 we found a decrease of antibody levels. There was a significant difference in anti-SARS-CoV-2 decrease between groups of patients according to disease severity, with higher reduction of IgG levels in severe patients (Table 3).

## Discussion

This is, to our knowledge, the first report analysing the Vit. D status and magnitude of anti-SARS-CoV-2 IgG antibody production in COVID-19 patients. Although in our study baseline levels of Vit. D were below normal values in 65.4% of patients, these values were in agreement with other publications^36–38^. We show that Vit. D distribution was affected by age and disease severity, but not sex. Similar findings were described in several other studies^39–42^. Ilie et al. also showed a negative correlation between Vit. D levels and COVID-19 cases and mortality^8^. A meta‐analysis with a total of 1368 COVID‐19 patients also showed that low Vit. D levels were significantly associated with poorer patient outcome and prognosis^42^. In contrast Hastie et al. found no link between Vit. D and risk of severe COVID-19 infection and mortality in 341,484 UK Biobank participants^43^.

Vit. D has well recognised immunoregulatory actions; it reduces production of pro-inflammatory cytokines (IL-6, IL-8 and IL-17) and increases anti-inflammatory cytokines (IL-10) leading to down-regulation of TH1 cells and up-regulation of TH2 cells^14^. It is therefore assumed that VDD could be a central factor in ‘cytokine storm’ seen in COVID-19 infection; patients with low concentrations of Vit. D (≤ 30 nmol/l or ≤ 12 ng/ml) have demonstrated significant, elevated markers of cytokine storm^44^. In this study we found a significant negative correlation between Vit. D levels and various inflammatory markers including IL-6, CRP, ESR and D-dimer. Despite observing trends, albeit not significant, Carpagnano et al. found higher levels of IL-6 in COVID patients with severe VDD^45^. IL-6 also plays a key role in cytokine storm and induces rise of CRP^46^, a well known inflammatory marker that also significantly increases in severe forms of COVID-19^47,48^. Since IL-6 bioactivity may change in the presence of Vit. D, CRP may be a more accurate indicator of pro-inflammatory cytokines than IL-6^46^. Several studies, which are in line with our study, have shown an inverse association between Vit. D and CRP^14,21^.

In COVID-19, virus-specific B-cell mediated humoral immunity has been implicated and majority of patients seroconverted during recovery phase. In this study 97% of patients seroconverted with higher IgG levels in males than in females, but with no significant difference. The same results were found by Kutsuna et al. but they reported a significant difference for the fact that in that study significance was set at p = 0.1^32^. Also Robbiani et al. reported higher anti-SARS-CoV-2 IgG in men than in women^49^. Age distribution of anti-SARS-CoV-2 IgG in this study was significantly different between the groups, with higher levels in patients > 50 years. This finding is in line with other studies^50,51^.

Our results confirm previous findings that clinical COVID-19 disease severity is associated with higher anti-SARS-CoV-2 serum-IgG antibodies^27,32^ although To et al. found that elevated antibody titers do not correlate with the severity of disease^52^. The exact immune mechanisms responsible for different IgG responses between different forms of disease are not known. Whereas Gozalbo-Rovira et al.^18^ reported that patients with severe forms of the disease could be exposed to higher and more perdurable viral burdens, Hoepel et al. suggest that worsening of disease during SARS-CoV-2 infection could be caused by antibodies^53^. Gao et al. found that moderate and severe symptomatic patients exhibited a significant increase in frequencies of B-cells compared to healthy controls^54^. This demonstrates that compared with potent SARS-CoV-2-specific B cell responses mounted in COVID-19 patients after moderate or severe illness, asymptomatic or mild symptomatic COVID-19 patients only induced weak and transient SARS-CoV-2-specific B cell responses^54^.

By analysing anti-SARS-CoV-2 IgG in relation to comorbidities, significantly different distribution was found among patients with and without diabetes but not with and without hypertension, results similar to Kutsuna et al.^32^. Esperança-Martins et al. found significantly lower levels of anti-SARS-CoV-2 IgG in cancer patients^55^, a finding we could not corroborate possibly due to the low number of cancer patients recruited in the study. With regard to therapies, our finding that corticosteroid use and IgG-w4 were associated were similar to findings by Kutsuna et al.^32^. This association may be a result of the use of corticosteroids in moderate and severe patients, which already showed a relationship with IgG levels.

In this study we sought to determine whether the magnitude of SARS-CoV-2 response was related to an inflammatory state. Correlation analyses show that anti-SARS-CoV-2 IgGs were significantly correlated with IL-6, CRP, ESR, LDH, lower WBC count, higher persentages of granulocytes, lower persentages of lymphocytes and lower persentages of monocytes. Gozalbo-Rovira et al. also found weak or very weak correlation of anti-RBD-IgG with inflammatory markers such as CRP, IL-6, D-Dimer and LDH^18^. Corrrelation between anti-SARS-CoV-2 antibodies and CRP levels were reported also by other authors^32,56,57^. Correlation of anti-SARS-CoV-2 antibodies with LDH levels found in first week PSO and maximum values during the disease are in line with Kutsuna et al.^32^. Increase in neutrophil count and decrease in the lymphocytes were more common in severe cases than in moderate cases of COVID-19^58,59^ and indicate the intensity of inflammatory response. In this study we found that the maximum values of granulocytes and minimum values of lymphocytes during the course of disease are in correlation with anti-SARS-Cov-2 IgG which are olso found to be higher in severe cases of disease who also showed higher inflammatory responses. The same was found by Gozalbo-Rovira et al.^18^.

A key question of this study was if baseline Vit. D levels may influence the serological response in patients with COVID-19. Similar to our findings, Yonghong et al. in 18,148 individuals found that SARS-CoV-2 seropositivity was not associated with having a Vit. D level less than 30 ng/ml before or during the pandemic independently of other risk factors^38^. Also Barassi et al. reported that there was no relationship between Vit. D and anti-SARS-CoV-2 IgG values^60^. Although Kaufman et al. found strong and inverse association between Vit. D and SARS-CoV-2 positivity, in contrast to our study they did not carry out quantitative analyses between these parameters^35^.

Prior studies reported that SARS-Cov-2 IgG levels decline during time post infection^61,62^ and the same was found in this study in which in 65% of patients with measured IgG-m4, levels of IgG decreased after three months. A significant difference in decrease of SARS-CoV-2 IgG was found between groups of patients according to disease severity, with higher reduction of IgG levels in severe patients. Our results are in line with the results of Kutsuna et al. in which in moderate and severely ill patients titers of IgG tended to decline 60 days PSO compared to mild cases^32^. Ma et al. analysed decline rate of IgG and predicted convalescent patients’ SARS-CoV-2 IgG to be undetectable approximately 273 days after hospital discharge^63^. This study has some limitations. First, the sample size of this study is modest and not all patients had all laboratory analyses completed making paired analysis difficult. The second limitation of the study was the collection of samples at different sites.

## Conclusions

In summary, anti-SARS-CoV-2 IgGs and Vit. D shares common parameters associated with inflammatory state. This could lead us to suppose that Vit. D signaling, targeting several immune-mediated pathways, protects against severe forms of COVID-19 but it does not directly affect the anti-SARS-CoV-2 IgG production. However, further work needs to be completed to address whether Vit. D influences serological response in COVID-19 patients.

## Methods

### Study population

COVID-19 patients (n = 69), diagnosed by RT-PCR analysis of nasopharyngeal swab, were enrolled in this prospective study. Patients were either hospitalized at the University Clinical Center of Kosova (n = 42) or at the outpatients clinic (n = 27). Of these patients, 52 were recruited in the first week post-symptom onset (PSO); the remaining (n = 17) were recruited 3 weeks PSO. Fifty patients in this study group are also part of the research project "Association of Il-6 and other biomarkers of inflammation with outcome of Covid 19 patients: A study from Kosova”.

In an attempt to determine the relationship between the magnitude of anti-SARS-CoV-2 IgG levels with Vit. D and the “inflammatory state” of patients, we analysed anti-SARS-CoV-2 IgG in relation with Vit. D and inflammatory biomarkers. For anti-SARS-CoV-2 and Vit. D measurement blood was collected from the cubital vein. Serum was separated from whole blood (4 ml) collected in granules and clot activator tubes and stored at – 20 °C until measurement of Total 25(OH)D and anti-SARS-CoV-2 IgG antibodies.

### Measurement of total serum 25(OH)D

Total serum 25[OH]D was quantified using an electrochemiluminescence assay, (Roche Cobase 411) according to the manufacturer’s instructions. Levels obtained were divided into three categories according to Endocrine Society Clinical Practice Guideline on Vitamin D: (1) deficient (≤ 20 ng/ml), (2) insufficient (21–29 ng/ml) and (3) sufficient (≥ 30 ng/ml). Vit. D was measured twice; in week 1 since symptom onset (Vit. D-w1; n = 52) and week 4 since symptom onset (Vit. D-w4; n = 50, two patients passed away without second measurement of Vit. D).

### Measurement of anti-SARS-CoV-2 IgG antibodies

Anti-SARS-CoV-2 IgG antibodies levels were determined using the SARS-CoV-2 IgG II Quant assay, an quantitative chemiluminescent microparticle immunoassay (CMIA), (Abbott) according to the manufacturer’s instructions. The cut-off value for test positivity was 50.0 AU/ml with analytical measurement interval of 21–40,000 AU/ml. In 67 of 69 patients (as mentioned above, two passed away before week 4) anti-SARS-CoV-2 IgG antibodies were measured at week 4 since symptom onset (IgG-w4; n = 67). In 17 of these patients, IgG levels were also measured at 4 months since symptom onset (IgG-m4; n = 17).

### Clinical parameters

Additional information was collected from recruited patients including, age, sex, illness severity, comorbidities, medications used to treat illness, clinical outcome, inflammatory markers (IL-6, CRP, D-dimer and LDH), hematological parameters [Erythrocyte Sedimantatio Rate (ESR), White Blood Cell count (WBC), percentages of granulocytes, lymphocytes and monocytes and platelet count] during acute phase of COVID-19.

### Statistical analysis

Data were tested for normality by means of Kolmogorov–Smirnov and Shapiro–Wilk test. Categorical variables were presented as frequencies and percentages, whereas skewed continuous variables were expressed as medians and inter-quartile ranges. Chi-Square test was used to examine relationships between categorical variables. Due to non-normal distribution of data, comparisons across disease severity categories and age groups were conducted using Kruskal–Wallis test; Mann–Whitney U Test was used for comparisons accross two categorical independent goups (sex and hospitalisation); whereas comparison accross positive and negative ranks was carried out by using Wilcoxon Signed-Rank Test. Spearman’s rank correlation coefficient was used to gauge monotonic relationship between Vitamin D and IgG levels, as well as inflammatory and hematological markers. Statistical analyses were carried out with SPSS (version 25) and/or Graphpad Prism v9.0. All tests were 2-talied and P-values < 0.05 were considered significant.

### Ethics declaration

This study was approved by the Ethics Committee at University Clinical Centre of Kosova (reference no 2548/2020). Written informed consent was obtained from all participants, in accordance with the Declaration of Helsinki.

## Data Availability

The raw data supporting the conclusions of this article will be made available by the authors, without undue reservation.
